# Supporting children who have a parent with a mental illness in Tyrol: a situational analysis for informing co-development and implementation of practice changes

**DOI:** 10.1186/s12913-020-05184-8

**Published:** 2020-04-19

**Authors:** Ingrid Zechmeister-Koss, Melinda Goodyear, Heinz Tüchler, Jean Lillian Paul

**Affiliations:** 1 Ludwig Boltzmann Institute for Health Technology Assessment, Garnisongasse 7/20, 1090 Vienna, Austria; 2 School of Rural Health, Monash University Melbourne, Wellington Rd, Clayton, Victoria 3800 Australia; 3 Mental Health Research Group Programme, The Village, Ludwig Boltzmann Gesellschaft, c/o MedUni Innsbruck, Tirol Kliniken GmbH, Schöpfstraße 23a, 6020 Innsbruck, Austria; 4 Department of Psychiatry, Psychotherapy and Psychosomatics, Division of Psychiatry I, Medical University of Innsbruck, Anichstraße 35, 6020 Innsbruck, Austria

**Keywords:** Mental illness, Children, Situational analysis, Context analysis, Implementation science

## Abstract

**Background:**

A research project, which aims to improve the situation of children of parents with a mental illness (COPMI) is currently underway in the Austrian region of Tyrol. The project aims to strengthen formal and informal support structures around the child, through enhancing their village of collaborative support. Understanding the current situation in the region is vital for implementing practice change. This paper aims to gain knowledge regarding the Tyrolean societal and service provision context.

**Methods:**

We collected qualitative (17 interviews among stakeholder and people with lived experience) and quantitative data (e.g. health insurance data) regarding overall societal characteristics, epidemiology of mental illness, currently existing services, uptake of services, and current practices and challenges of identifying and supporting COPMIs. We analysed data along eight external context dimensions: 1) professional influences, 2) political support, 3) social climate, 4) local infrastructure, 5) policy and legal climate, 6) relational climate, 7) target population, and 8) funding and economic climate.

**Results:**

We identified that there is awareness of potential challenges related to COPMIs at both a professional and planning level. Additionally, there is a lack of installed support processes and standards to meet these children’s needs across Tyrol. A variety of services are available both for unwell parents, as well as for families and individual family members. Yet, only one small service addresses COPMIs directly. Services fall into different sectors (education, health, social affairs) and are funded from different sources, making coordination difficult. Access varies from universal to rather restricted (i.e. through referral). The potential number of parents which could be reached in order to identify their children via adult mental health, differs considerably by setting. Societal structures indicate that the informal and voluntary sector may be a realistic source for supporting COPMIs.

**Conclusions:**

The societal structures and the current services provide a rich resource for improving identification and support of COPMIs, however considerable coordination and behaviour change efforts will be required due to the fragmentation of the system and professional cultures. The insights into the context of supporting COPMIs have been of high value for developing and implementing practice changes in the local organizations.

## Introduction

Research has shown that approximately 25% of children worldwide live with a parent who has a mental illness [1–6]. This paper focuses on children of parents with a mental illness who will be referred to as ‘children’ or ‘these children’ for the remaining of the paper. These children have an increased likelikhood to experience additional adversities due to their family circumstances, which for some, may lead to negative long-term difficulties, in addition to substantial lifelong impacts for individuals, governments and the wider community [4, 7–12]. These childen often remain invisible to the community or professional services, with many barriers to early identification, especially in the (adult) mental health and social care settings [13–15]. Consequently, children’s needs often remain unmet, and they may not have the opportunity to access support. The situation can be further exacerbated due to limited coordinated and collaborative care, which could otherwise enhance the provision of formal and informal support for children and their families [16].

Several initiatives around the world have addressed this situation by developing specific interventions and / or policies [17–22, 14, 23, 24]. Existing approaches usually focus on one selected target group (parent vs child), setting (psychiatry vs community), and/or diagnoses or age groups. Our project ‘How to raise the village to raise the child? Supporting children who have parents with a mental illness in Austria’ instead develops a comprehensive, multisector approach for multiple family members, regardless of parental diagnosis or child age [25]. For further information on the project see www.village.lbg.ac.at. The four-year project is implemented by an international and interdisciplinary research team in the Western Austrian pilot region of Tyrol, focussing on early intervention, based on evidence that early intervention can work [19, 26, 27]. We approach early intervention through evidence-informed co-development (together with stakeholders), implementation, and evaluation of two practice approaches: (1) improving identification of children via parents in adult (mental) healthcare, and (2) strengthening child-focused support networks. The former practice we define as ‘sensitive screening’ (SENSE), and the latter as ‘collaborative village approach’ (CVA). The co-development of SENSE and CVA practices, completed in early 2019, included: key service providers, service planning representatives, and people with lived-experience from the Tyrol region. The group participated in a series of design workshops, held over a six-month period. Professionals included management, as well as line staff, from psychiatric/psychotherapeutic and social care services across the region, supporting adults and/or children, with interdisciplinary professional background.

Principles underpinning SENSE and CVA practices include child empowerment and participation, the ‘child’s voice’, strength-based approaches, and collaborative care. According to the project concept, for installing the support network within the CVA, informal support sources will be activated as a primary source, and supplemented by formal support, where needed. The project draws on community-capacity building approaches, developing a supportive network of allies around a person [25]. The entire process is supported by a ‘competence group’, consisting of seven young adults who grew up with a parent with a mental illness. They meet with the research team monthly and provide active participation in various elements of project activities.

According to the definition by Craig et al. (2008 and 2019) [28, 29], the practice approaches, SENSE and CVA that are planned to be implemented, can be defined as a complex intervention: (1) there are a number of interacting components, (2) there is a certain degree of difficulty of behaviours required by those delivering or receiving the intervention, (3) a high number of groups or organisational levels are targeted by the intervention, (4) there are a number of different outcomes, and (5) a degree of flexibility or tailoring of the intervention exists. It has been argued that complex interventions may unfold differently in different contexts [30] and replicating them in different jurisdictions without modifying according to context often leads to disappointing results [31]. Evidence from complexity and implementation science strongly suggests that external factors, governing practice within the service context, may be a major driver or barrier for successful implementation [32–34]. Consequently, a good understanding of the current practice, service utilisation, and the existing needs in the Tyrolean pilot region, is a pre-condition for developing and implementing the planned practice changes. Furthermore, understanding the context is an important step in developing logic models of the practices changes [35], another important step of the co-development process within this project.

The aim of this paper is to summarise the results from a multi-dimensional situational analysis, in order to obtain an in-depth understanding of the local context and the existing needs for supporting the affected children and their families. The knowledge gained from this process informed the design of the subsequent co-development process, including the practice approaches SENSE and CVA for identifying and supporting the affected children.

## Method

### Conceptual framework for analysis

**Fig. 1:**
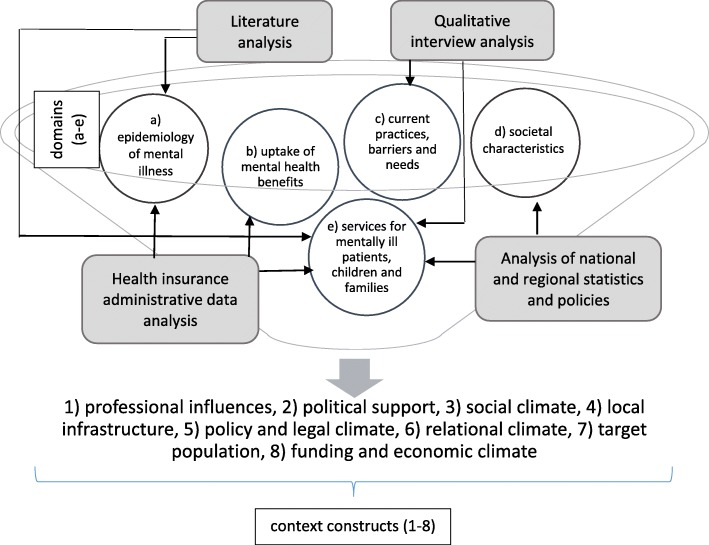
Conceptual framework and empirical data sources for analysis

Figure 1 depicts the conceptual framework for the situational analysis. We structured our analysis along analytical categories, based on Watson et al. (2018) [32] who recently developed a conceptualization of the external context influencing implementation of practices in health and social care. To derive empirically observed external context factors, they conducted an iterative literature analysis, and an inductive thematic content analysis. Resulting from this work, they developed homogenous, well-defined, and mutually exclusive context categories. We selected this framework, which includes an exhaustive list of external context factors, to identify factors that may impact the implemention of our own practice approaches in a structured way, an approach supported by authors own observations [32]. The taxonomy consists of eight context constructs (see Table 1): (1) professional influences, (2) political support, (3) social climate, (4) local infrastructure, (5) policy and legal climate, (6) relational climate, (7) target population, (8) funding and economic climate ([32], p. 6).

**Table 1:** Framework for context constructs

Context construct	Definition
professional influences	formal or informal norms, rules, policies or standards guiding the professionalization of individuals involved in the implementation
political support	extent of backing from public officials or special interest groups
social climate	beliefs, values, customs and practices of the larger community and / or system within which the intervention is embedded
local infrastructure	physical, technical, service, and training structure or resources existing in the community or larger system in which the intervention is embedded
policy and legal climate	formal national, state, community, or system regulations (rules, policies, laws) impacting the intervention
relational climate	degree and / or quality of relationships with external entities (e.g. referral sources, partner organizations, regulation agencies, etc.) not involved in implementation but key to successful intervention delivery
target population	characteristics associated with individuals the intervention is designed to impact including population needs, culture, beliefs, preferences, locatability, ability to access, and motivation to engage
funding and economic climate	the character of the national, regional, or local economy and availability of funding as related to the intervention

Source: Watson et al. (2018) ([32], p. 6)

To describe the eight context constructs in Tyrol we collected empirical information on five domains: (a) epidemiology of mental illness in Tyrol, (b) uptake of mental health services, (c) current practices and challenges of identifying and supporting affected children, (d) overall societal characteristics (demographic, socio-economic and cultural dimension), and (e) services that currently exist within adult mental health care and for supporting children, parents and families as a whole (Fig. 1).

### Data sources

Our empirical material included one primary data and a variety of secondary data sources: The former were interview transcripts from 17 semi-structured qualitative interviews, conducted with adult expert stakeholders in Tyrol. The interviewees were selected by purposive sampling to achieve maximum diversity in terms of relevant sectors, service levels, providers and professions for supporting children who have a parent with a mental illness and their families, and people with lived-experience. Regarding people with lived experience, no restrictions other than age were defined (i.e., people could either be a parent with a mental disorder or an adult who grew up with a parent with a mental illness, or both). Specific research to understand the current situation for children under 18 years in Tyrol is treated seperately within a dedicated ‘child voice’ program of the research [25]. The categories (which sectors, which professions etc.) for selection were based on a comprehensive service mapping [36]. Interviewees represent all relevant sectors: healthcare (8), social care (6), educational (2) and the informal care sector (1) and within healthcare adult mental health (4) and child /adolescent mental health (1), as well as inpatient (2) and outpatient services (3). Secondly, we approached interviewees within service planning (2) and from the different types of service providing organisations (13). Among the latter, interviewees were identified as either actively working with families, parents or children. Additionally, we interviewed people with lived experience (2; one was an adult COPMI and one was both a former COPMI and a parent with a mental illness). Eight interviewees were male and nine were female. They represent the disciplines / professions of social work, psychology, medicine, social paedagogy and social science. Participants were approached via telephone or email. Most of them agreed to be interviewed in person. One referred us to a colleague. The location was selected based on the interviewee’s preferences. Most of them were conducted face-to-face in participants’ workplaces, and the remainder (including those with people with lived experience) were conducted in the project’s office space. Four were telephone interviews. The interviewers had no previous professional relationships with the interviewees except in one case. None of the interviewers had working experience in interviewees’ practice fields. All of the interviewers were female. Two interviewers were from Austria and had minimum or advanced knowledge on the Austrian welfare state structure. The remaining ones (three from Germany and one from Australia) had less knowledge on the Austrian care and support structures. In some of the interviews student assistants accompanied the interviewer. The interviews lasted on average 45 min and transcripts were returned to the participants for comments and / or corrections. The interviews addressed the current practice for supporting children and their families, as well as perceptions of required workforce changes and barriers. They were conducted to prepare the co-development and implementation process. Findings addressing the aims of this current paper are included. The interview guide used is available in the supplementary file 1 and has so far not been published elsewhere.

There were two types of secondary data sources used. Firstly a ‘COPMI-mapping report’ [36] which summarises published national and regional statistics and (policy) papers (identified by hand search and regional expert consultation) aiming to obtain a broad understanding of the current service patterns, and to identify all types of services within the health, social and educational sector in terms target groups, eligibility criteria, geographical distribution, financing and governance structures, etc. It maps available in-kind, as well as cash benefits, whereby the latter refers to monetary transfers within the health and social care system that Tyroleans may claim. Secondly, we used our recently published report on mental health service uptake based on an analysis of Tyrolean health insurance data (claims data) [37]. The report covers a range of essential mental health benefits: hospital inpatient, day care, and inpatient rehabilitation services according to an ICD-10-F diagnosis (psychiatric diagnosis according to the international classification of diseases [38]), outpatient psychiatrist specialist services (adult and child/adolescent specialists), psychotherapy services, psychological services, prescribed psychotropic drugs, and sick-leave according to an ICD-10-F diagnosis. Details on the data, analysis methods, data cleaning and quality checks, and limitations of both secondary data sources have been described and published elsewhere [36, 37, 39]. Furthermore, for all types of secondary data used, translation of German contents into English was done by the first author (IZ) using a glossary of terms and institutions [40].

### Methods of empirical data analysis

Statistical methods were applied to analyse quantitative raw data (administrative data) and were descriptive, including counts, percentages, and ranges, depending on the type of data. Published quantitative data (e.g. national statistics) was used as presented, without any further data processing. Qualitative data from local stakeholder interviews were deidentified, transcribed, and analysed using iterative inductive content analysis to generate major themes [41]. Interviews were transcribed verbatim. In those cases where interviews were held in German, transcripts were translated into English by research team members who had profound knowledge of the system as well as high-level English language skills (AEP, ES, JK, HK). Each translated transcript was double checked within the team and uploaded into QSR International NVivo (Version 12) [42]. The first stage of data analysis involved reading each transcripts to become familiar with the data. Minimal notes were taken at this stage. Stage two involved an open coding process, line by line, using an inductive thematic coding technique directly into QSR International NVivo (version 12). To ensure transparency and reliability, coding was iterative with codes refined to become more focused, integrating initial categories, and catergories discussed and refined. Each transcript was double coded by at least two team members. Double codes were compared and discussed upon completion of the analysis. The research team met on several occasions to discuss any differences in themes and consensus was reached when all parties agreed on the category and its definition. Where translated quotes will be presented to support interpretations we tried to maintain the intention as much as possible, which may possibly be at the expense of correct grammar.

### Data synthesis

Figure 1 represents the way in which different data sources have been combined to populate the domains, and finally inform the eight context constructs. In other words, from all data sources included, we selected information that describes the five domains outlined in Fig. 1, being aware that most sources provided further information that goes beyond the scope of this paper. The five domains were populated in the following way: The regional epidemiology of mental disorders (a) was informed by our secondary data sources (published literature as well as by the administrative data analysis). Administrative data were further used to analyse the uptake of mental health services (b). Our interview data were used to gain information on current practice barriers and needs for identifying and supporting our target group (c). Descriptive statistics of published key socio-demographic and socio-economic indicators (again part of the secondary data pool) were used to gain knowledge on the overall societal characteristics (d). Finally, we used published national and regional statistics, (policy) papers out of the secondary data pool, supplemented by information from interviews to map existing services for mentally ill parents, their children or families in general (e).

## Results

### Professional influences

Practitioners interviewed from a wide variety of fields highlighted that there is a lack of routine identification of parents and their children, other than to check child care arrangements when a parent is hospitalised. Various reasons were given as to why professionals do not ask about children or the family circumstances. Some participants felt that addressing parenting issues might be seen as beyond professional responsiblities within adult mental health care. Therefore, if it happens is dependent on individual practitioners’ motivation rather than standardised processes or guidelines.

*“My impression and my knowledge is that it [whether patients have children] is very rarely asked and that it depends on whether someone is interested or not. I mean, if a doctor who has children of her own, she might - so, I’m just assuming from experience now - ask, do you have children? But it is not a standard.”* (interview 14).

*“It’s [asking about about children] not part of the system …*. *It’s not standard and maybe we could develop a standard as part of the project.”* (interview 1).

*“Very often they do their standard program and maybe in their standard routine it is easier to just look at the individual person and to see which kind of medication the person needs and what else does they need. To like try to ignore everything around because it makes things more complex and difficult and it means an additional workload.”* (interview 10).

Another reason mentioned by participants was the lack of education in medical training on the topic, which led to uncertainty as to how to approach the topic with patients.

*“Point one. It is simply not an issue in training for psychiatrists.”* (interview 1).

*“I think that it [asking about children and family circumstances] remains the biggest challenge in this whole subject area. It often is simply still too little information there and great uncertainty in handling it and IF and HOW to address it, that is simply still difficult.*” (interview 8).

Services outside adult mental health also show relatively poor guidelines and a lack of standardisation of documentation protocols to identify or support the children themselves. Lack of awareness and training, such as reflected in the school example below, may contribute to this specific professional behaviour.

*“Teachers might just see that the performances is going down. You need to have – the kind of values …*. *I think very important is the way you look if you see a person with empathy and you look ‘what is going on with you – why you miss school?’.”* (interview 12).

*“But it definitely needs to be involved in the training of teachers. I think it would be great if the training for the prospective teachers was optimized and such content is brought in.”* (interview 3).

Importantly, while individual professionals have begun introducing a family-focused care philosophy, supporting the parent in their parenting as a principle of care has not been implemented as care standard.

*“It is often very difficult that adult psychiatry [in Tyrol] is not interested [in a systemic perspective]. That is strange for me because I think just in the interest of the client it should be from a systemic perspective.”* (interview 10).

*“The context of taking care of the children is one thing, but what I do notice, what exists even less is a systematic support of the parents in relation to this topic, parenting and this whole topic of guilt and support possibilities of the parents.”* (interview 12).

Participants described that even if they identify severe problems for children in their daily funcitoning, the adult mental health team usually does not get into contact *with* the children directly or talk with the parent about child issues, but the traditional way of work is to communicate with different institutions *about* the child.

*Interviewee [after interviewer asked if adult mental health practitioners get into contact with the children if they identify a caring need]: “Well, not really with the children but with the institutions outside [such as child and youth welfare].”* (interview 7).

### Political support

The mapping of the Tyrolean situation revealed several self-help associations and programs which have a mental health focus. Some are national with regional groups in Tyrol, while others are Tyrol-specific [36, 43]. One directly targets relatives of people with mental illnesses. However, during the interviews, it became apparent that while they often deal with individual cases involving children who have a parent with a mental illness, the interest group has not achieved system changes that would improve the situation of dependent children who have a parent with a mental illness.

*“We advised relatives what they could do with the children. But we didn’t accomplish … that we from our self-help association achieve something very concrete for the children. I’m still sorry today.”* (interview 14).

The mapping exercise also demonstrated that within public administration at the regional level, there is a mental health coordinator employed by the Tyrolean government. This may play a role in supporting workforce development on the regional governmental level. Mental healthcare coordinators develop and coordinate patient-oriented mental healthcare and provide care for people with addictive disorders. Services cover outpatient and inpatient care, rehabilitation and psycho-social services, prevention and self-help activities. Additionally, the coordinator organises a steering committee, including Tyrolean psycho-social care providers and advises the regional government and the Tyrolean hospital fund. Yet, as revealed in the interviews, the topic of supporting children who have a parent with a mental disorder has not been systematically addressed at the strategic level so far.

*“Yes, it [the topic] comes up because it can be said that vulnerable children and young people who are in contact with the psychiatric landscape usually have social problems, anomalies, homelessness, alcoholism or violence and so on in the background. It [the topic] comes up all the time. But it’s not a priority right now.”* (interview 1).

### Social climate

Since one key component of our intervention is to activate informal care to support the children [25], the social capital of our pilot region may play a crucial role for implementation. Considering demographics and socio-economics within Tyrol, the region can be classified as traditional and conservative in relation to family formations and characteristics of education/employment [36]. For example, from around 140,000 dependent children between 0 and 18 years (19% of the Tyrolean population), the vast majority lived in dual-parent families [44, 45]. Additionally, there was a slightly higher number of children per household than the Austrian average [44]. 30% of the employees were working part-time, the majority of whom were women [46]. In 2016/2017, most children were cared for at home, with only 33% of children aged 0 to 14 years were using child care in Tyrol [47].

Likewise, traditional gender roles were expressed in the interviews. When interviewees reflected on current needs, support needs are primarily identified for mothers ( “… *and especially the sick parents need support and especially the mothers*”/ interview 14) while fathers are not mentioned (“*they [outreach services] can support above all the mothers (..) in their educative ability”*/ interview 14).

However, a difference between rural and urban areas was described.

*“Here it is even more archaic - archaic mechanisms are still in progress. There are also many large families here. I think that for many generations - dealing with problems, feelings, women’s dedication to the family and such things. These are still very, very much more pronounced here than, for example, in the city.”* (interview 12).

Regarding religion – another likely influence on social climate – a catholic denomination plays the most important role in Tyrol (~ 80% of population) [48]. Furthermore, the political history shows that the conservative people’s party has always had an absolute or relative majority in the regional government [49]. Another social climate factor is citizenship, and within Tyrol, 85% of the population are Austrian citizens. There is a Turkish community and there are people from South-Eastern European countries living in Tyrol, however, the largest groups with non-Austrian nationality are Germans and those with similar cultural background [50].

### Local infrastructure

**Fig. 2:**
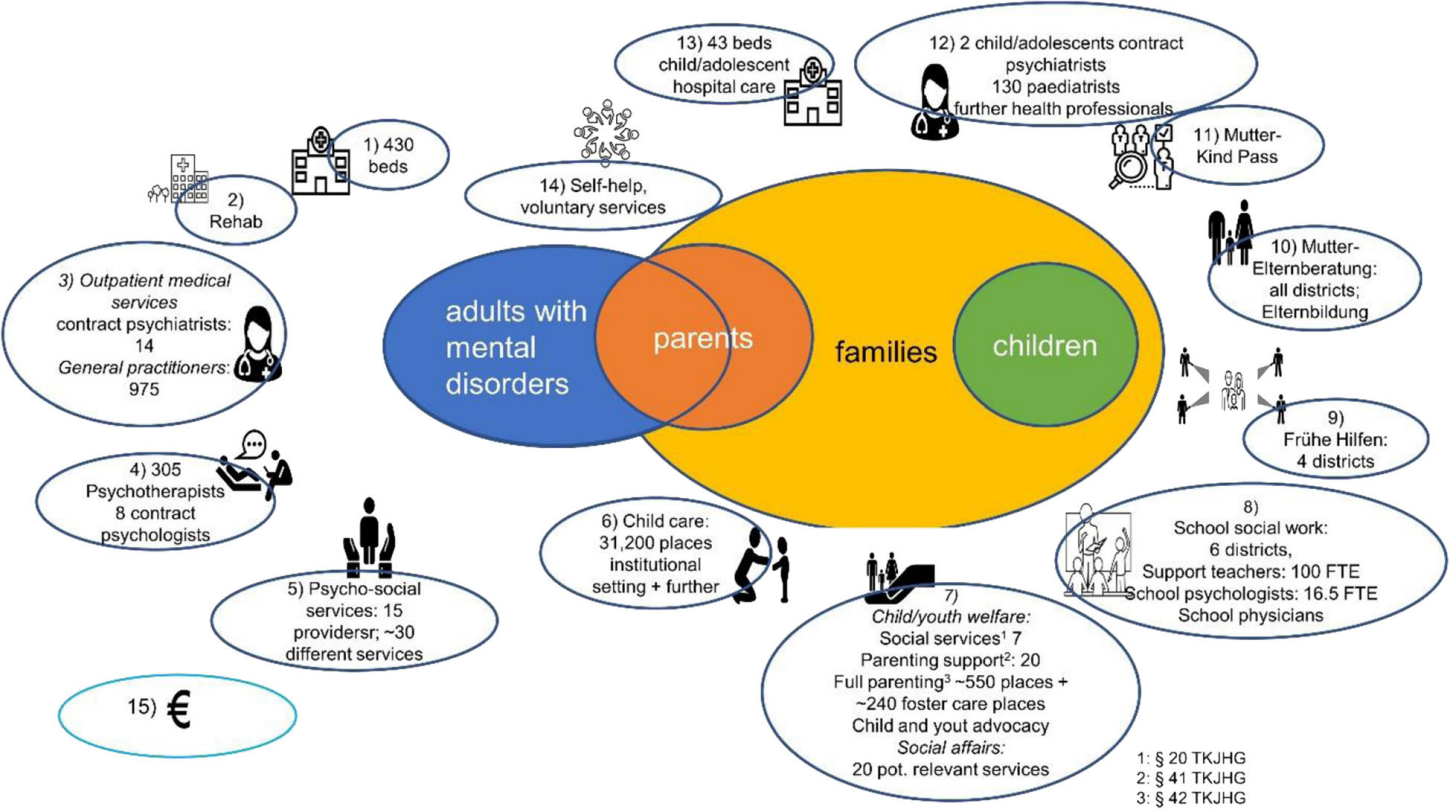
Overview on available services. 1: Hospital services (inpatient, day care, outpatient); 2: inpatient rehabilitation, 3: outpatient services (psychiatrists, general practitioners, other health professionals such as occupational therapists, etc.), 4: psychotherapists and psychologists, 5: psycho-social services (regional government), psychological counselling (health insurance), psycho-social counselling (regional government); 6: child care; 7: child and youth welfare and social services; 8: services in schools (school social work, school physicians, school psychologists, support teachers); 9: Frühe Hilfen (early intervention in pregnancy and early childhood); 10: parental services, 11: Mutter-Kind Pass (screening programm in pregnancy and early childhood); 12: outpatient specialists for children and adolescents: child and adolescent psychiatrists, paediatrists, phsychotherapists ...); 13: hospital services for children and adolescents: child and adolescent psychiatry, paedeatric units; 14: self-help and services offered by voluntary workers, 15: cash benefits; only those services where data on capacities were available are quantified; for more details see [36]

We identified a broad range of potentially relevant services for identifying and / or supporting affected children (Fig. 2) [36]. The core services for identifying the children within the project scope are adult mental health services. They include a large variety of services (hospital and outpatient, as well as psycho-social, employment-related, and inpatient rehabilitation services) and are provided in various settings. Services beyond adult mental health care, which may also play a role for improving identification of the children in Tyrol, include the ‘Early Prevention Service’ for pregnant women and families with children up to 3 years (Frühe Hilfen), a screening program in pregnancy and early childhood (Mutter-Kind Pass), primary healthcare services, or services provided by specialists in schools including school psychologists, school social workers, or school physicians.

Following identification, a variety of services belonging to the child and youth welfare field were identified that may all be potentially relevant in supporting the children and families in their daily life. Additionally, there are a number of social services offering support for children and/or families which could be utilised by children of parents with a mental illness (e.g. adolescents / youth centres with specific meeting areas, coaching for adolescents, and support for girls). In addition, a number of services have been identified that offer general parenting support (e.g. parental counselling services provided by the regional government in each district). These services could be used for parents with a mental illness to support them in their parenting roles. As mentioned earlier, there are activities within the informal sector, most significantly, self-help groups specific to mental health (e.g. self-help groups for family members of people with mental disorders), and further initiatives in the voluntary sector such as ‘host grandmothers’. Out of all services that may be potentially relevant for supporting children, only one service identified (‘Kinderleicht’) specifically addressed children of parents with a mental illness, more specifically, children of parents with addictive disorders. However, this service is relatively small and at this stage, only available within two regions in Tyrol.

Besides in-kind services, cash benefits have been identified which could be accessed by families impacted by mental illnesses. Importantly, cash benefits which are Tyrol-specific and accessible in an emergency situation or for low-income families were identified (e.g. ‘Kinderbetreuungszuschuss des Landes Tirol’ supporting child care costs).

However, while the mapping data indicate that there is a broad variety of services available, the interview data revealed some obvious gaps in the care pathway for the children. This is for example the case in emergency situations, where individual solutions have been described to bridge the gap between time of admission and opening hours of child and youth welfare offices.

*“And sometimes we had the problem, especially during the nights. We knew there was a child at home and nobody can care for this child right now. And then we have to get in contact with the Department for Paediatrics, not psychiatry unit but the normal clinic, asking “Do you have a bed only for one night? We need a place for this child. We don’t know what to do now. There is nobody else who can care for this child now. Could you please take this child for one night?” But, also this is a very bad situation. I mean the child is very embarrassed about this situation, that somebody else comes at home again, takes the child, to come to the clinic and go there knowing nobody, only have to sleep here tonight, we’ll care tomorrow, when the institution is working again and we will find a place for you.”* (interview 7).

Additionally, a general lack of services for the target group has been described.

*“I already had the impression that many requests were from the parents or then separated parents who are mentally ill, who were asking to get some offers or support for the children, but we simply have no services to offer*” (interview 4).

*“I think the situation for children with parents with psychiatric illness is not easy because there are no specific services for them. This is my general view …*. *I mean we see this problem but we do not know how to support the children*” (interview 7).

Additionally, more types of services were available in urban than rural regions, demonstrating geographical variation and a potential shortage of services to address specific needs in more remote areas.

### Policy and legal climate

Two national policies (both launched in 2012) were identified that may be utilized for the implementation of our planned practice approaches in Tyrol. Firstly, the Austrian child and adolescent health strategy includes five strategic goals, which are all addressed by our planned practice approaches: improve equal health opportunities, strengthen and maintain individual health resources, support healthy development early on, reduce health risks, raise awareness for health in all policies [51]. Secondly, several out of the ten Austrian health targets [52] are closely linked to our planned practice approaches and the envisaged impact. For example, the practice approaches in the Village project target aim number five (to strengthen social cohesion as a health enhancer), particularly outlining the importance of social relationships and social networks, which will be addressed by the project’s aim to activate the social support network around the child. Furthermore, they also target aim number six (to ensure conditions under which children and young people can grow up as healthy as possible), thus addressing our target group directly; and target aim number nine (to promote psychosocial health in all population groups). In addition to tackling stigma, it is outlined that “people suffering from mental disorders, and their relatives (especially parents and children) need comprehensive, appropriate care services, and their (re) integration into society must be assured ([52], p. 15).”

### Relational climate

As outlined in Table 1, relational climate refers to the degree and / or quality of relationships with external entities (e.g. referral sources, partner organizations, regulation agencies, etc.) not involved in implementation but key to successful intervention delivery. In the mapping exercise, a vast variety of services were identified which may play a role in supporting children and their families. However, key to successful support will be to implement a coordination and collaboration process that is capable of delivering tailored support, according to individual families’ needs and involving all relevant actors.

As interviewees have observed, collaboration activities are already taking place, however, they seem to have only been established with some, but not all relevant organisations.

*“So, patients go to these institutions [support organisations for adults] when they leave from our clinic. But, of course when needed, they come back to the clinic, so we are very close to those institutions [support organisations for adults]. Not really with the institutions for children of mentally ill parents.”* (interview 7).

**Fig. 3:**
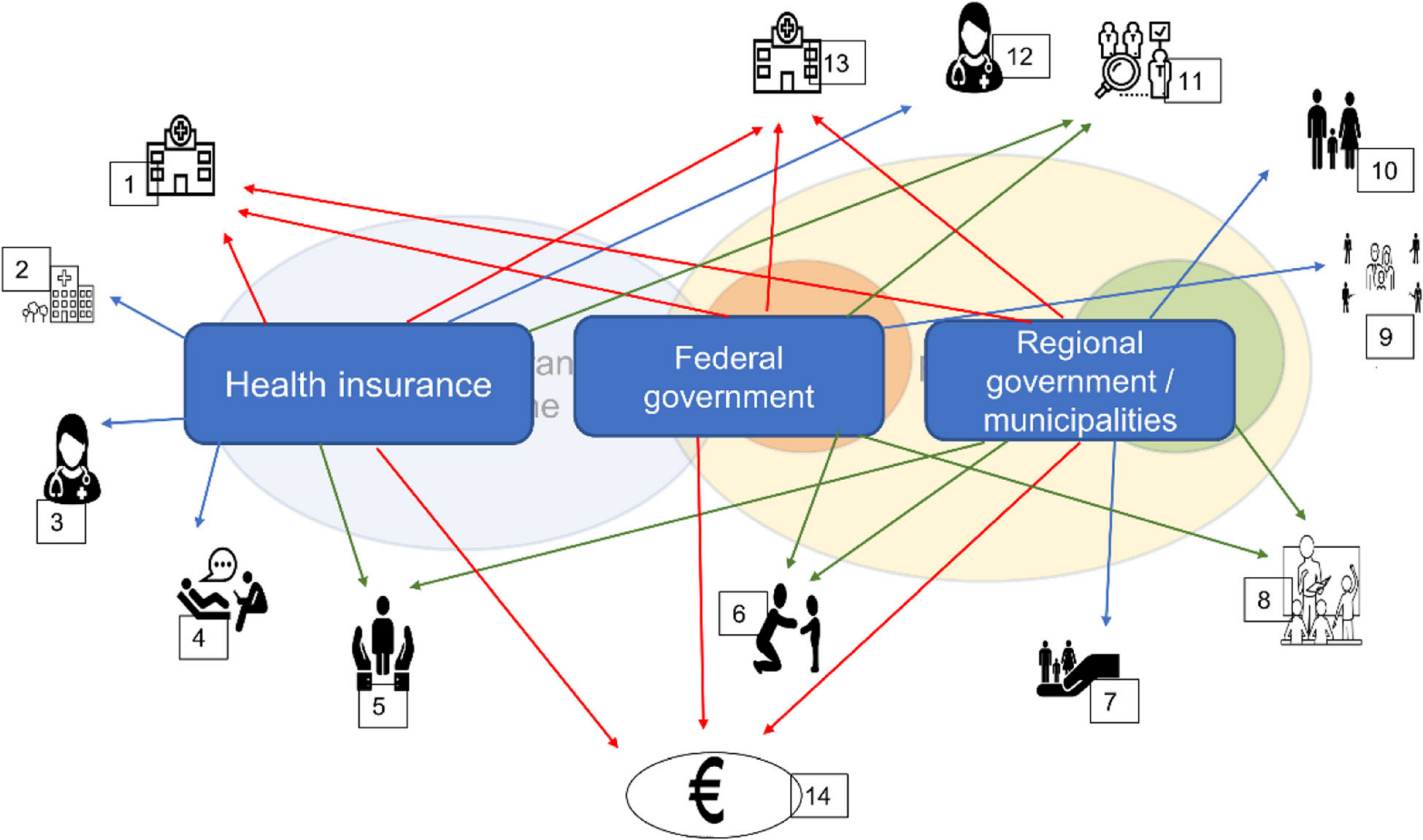
Financing structures for services. 1: Hospital services (inpatient, day care, outpatient); 2: inpatient rehabilitation, 3: outpatient services (psychiatrists, general practitioners, other health professionals such as occupational therapists, etc.), 4: psychotherapists and psychologists, 5: psycho-social services (regional government), psychological counselling (health insurance), psycho-social counselling (regional government); 6: child care; 7: child and youth welfare and social services; 8: services in schools (school social work, school physicians, school psychologists, support teachers); 9: Frühe Hilfen (early intervention in pregnancy and early childhood); 10: parental services, 11: Mutter-Kind Pass (screening programm in pregnancy and early childhood); 12: outpatient specialists for children and adolescents: child and adolescent psychiatrists, paediatrists, phsychotherapists ...); 13: hospital services for children and adolescents: child and adolescent psychiatry, paedeatric units; 14: cash benefits

The information from the mapping indicates potential causes for coordination barriers. According to the mapping results, services identified are legally assigned to different welfare state sectors, which makes coordination across sectors challenging. Most of identified services for parents with a mental illness are provided within the realm of the healthcare system. Yet, the majority of significant services for family, children, or parental support for their everyday life lie within the education or social affairs sectors. Consequently, families and service coordinators are confronted with a complex service financing system, whereby respsonsibilites can be borne by governments at federal, regional, or muniscipal level, or by the health insurance company, or a combination of these (Fig. 3). Regional government responsibility is central for many core services identified as supporting children’s daily life. Furthermore, compared to health services, funding of those social services is frequently ‘project based’, therefore subject to discretionary decisions and less sustainable [36].

Interview results added another layer to the coordination challenge. While experts welcomed the availability of a broad variety of services, this may also result in many different agencies involved with families, which could become a barrier for engagement and effective support.

*“The children are getting very confused. There are eight or six different caregivers working in [the] family. The children are overwhelmed with the situation and that is actually what we do not want.”* (interview 2).

As possible reasons, interviewees described limited responsibilities and competences of each single organisation, and competition between them.

*“They [these organisatons] say they only do this, they are only responsible for that, but when I need something, which perhaps needs a little bit of both, then I have to start again. I may need a third organization. That is the great difficulty, in our view, in supporting the families.”* (interview 2).

*“We need to move away from competitive thinking because there is enough work in the social and educational system for everyone”* (interview 3).

### Target population

**Fig. 4:**
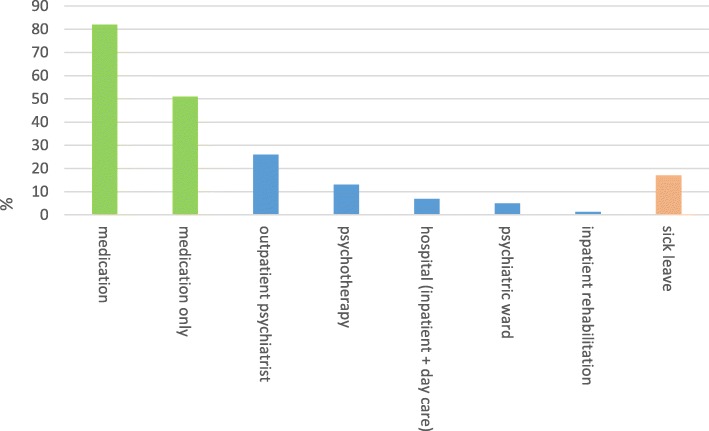
Use of benefits in 19–64 years old service users (*n* = 49,494); 2017. Use of more than one benefit per patient possible

The available data do not allow for identifying precise numbers of affected children or parents with a mental illness. Furthermore, robust epidemiological data on mental illness in Tyrol are lacking. However, the administrative data analysis [37] showed that ~ 50,000 insured persons aged 19–64 years (13% of the insured population) received some type of Tyrolean social health insurance (co)-funded mental health benefit. From those potential parents with a mental disorder, overwhelmingly, most were prescribed medication (82%), half of whom received medication exclusively, without accessing other types of insurance funded services (Fig. 2). Since general practitioners prescribe more than 90% of psychotropic drugs in Tyrol, [53] most people with a mental disorder who contact a professional service will at some point contact a general practitioner. People were most frequently prescribed anti-depressants (61%), followed by anti-psychotics (18%). Other types of psychotropic drugs were prescribed to less than 10% of patients. Forty percent of the 19–64 years old potential parents accessed some type of insurance-funded ‘physical’ service for mental health in 2017 (covering the hospital, outpatient services, or rehabilitation services). Services most frequently used were outpatient psychiatric services, accessed by over a quarter of this group, whereas only 7% were admitted to a hospital inpatient ward or received day-care treatment (Fig. 4).

**Fig. 5:**
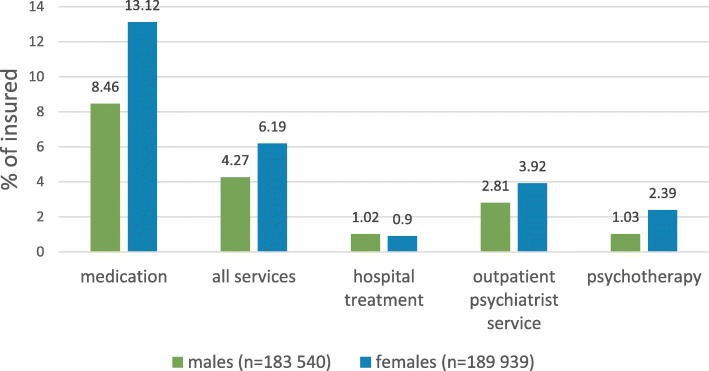
Gender characteristics of insured mental health benefit users (19–64 years)

A higher percentage of female insured persons aged 19–64 years used mental health benefits than male insured. This gender difference is particularly apparent for the use of medication, outpatient psychotherapy and psychiatrist specialist services, whereas the proportion is almost equal regarding hospital (inpatient and day care) services (Fig. 5).

Regarding hospitalised patients, the most frequently documented diagnoses were F1 (mental and behavioural disorders due to psychoactive substance use), F3 (mood [affective] disorders) and F4 (anxiety, dissociative, stress-related, somatoform and other non-psychotic disorders), whereby F1 was documented considerably more often in males than females, and F3 as well F4 were more frequent in female patients. Length of stay in hospital admissions varied considerably (range: 1 to > 300 days), however, 50% were discharged after 15 days. Furthermore, 50% of the patients were only admitted once during 2017 and a quarter of the patients were admitted in non-psychiatric wards (mostly internal medicine wards). No information is available on the frequency of use in services that are funded by other payers (e.g. psycho-social services which are funded out of regional taxes) or on the frequency of hospital outpatient service use.

The data on service uptake likely underestimate the true prevalence of people or parents suffering from a mental disorder. Expert interviews with service providers and people with lived experience revealed a number of barriers for families to seek help. Commonly, these children experience a high burden of care for their parents, but also secrecy and fear are paramount within these families.

*“The problem is … that the usual taboo mechanisms in families are still practiced. The problem is often in adult psychiatry the tendency is, in my experience, not to involve family members or the patient’s environment in that way, the parent, or even the children, to address this topic. Because, what I think is, that this is a taboo to talk about and promises difficulties. Yes. This is, of course, an extremely shameful story. Not to carry out the parental role, or to have harmed the children or such things...”* (interview 6).

*“In rural areas, we also notice that when we introduce new services, it takes longer for them to be accepted. … So that there is this barrier for saying, ‘I need support – [but] what do other people think of me then'? That’s still a topic.”* (interview 8).

Furthermore, a fear associated with child and youth welfare seems to be a major barrier for families to talk about their child in adult psychiatry. Stakeholders describe patients being reluctant to mention that they are parents in psychiatry settings or do not speak about the impact of the illness on parenting. They perceive that parents are worried that child and youth welfare will become involved and take away their children.

*“They keep saying child and youth office, although we are now called child and youth welfare, because it is the office that somehow has the bad taste that takes the children and never gives them back and so on. The barrier for mothers to go to child and youth welfare is often high.”* (interview 5).

According to the service mapping, available services in Tyrol are characterised by a high proportion of public funding. However, access to many of these publicly funded services is constricted via gate-keeping (e.g. particularly depending on referral from child and youth welfare) [36] or limited capacities of services (e.g. psychotherapy, childcare) [54]. Furthermore, many services are reserved for families and children with severe problems (e.g. serious neglect, abuse). Yet, as it was pointed out in the interviews, not all families may need professional intervention to help them manage the impact of mental illness, and professional support was even considered to be potentially harmful in cases where children are healthy.

*“Certainly, a third [of children] can arise relatively quickly from one’s own resources, i.e. where one can activate something in the family, something that has not been considered so far. A third, where you simply have to act a little more intensively, so that something happens. More or perhaps less a third is where you really say, “It really needs interventions now, there really is suffering among the children”, i.e. perhaps roughly 20%, where I really say, they need professional help. That is certainly not the case with all.”* (interview 13).

*“I am very concerned that these healthy children - for me these are healthy children - are diagnosed too quickly and receive some care too quickly, which may not be necessary for many. If the children experience a lot of normality and good social relationships in addition to the stress in the family, they can develop quite well and stay healthy. If they get diagnosed too soon, it means for them you’re not okay. Something’s wrong with you, you need something.”* (interview 14).

### Funding and economic climate

The availability of funding for the children is strongly interwoven with the system of social security. The Tyrolean system of social security is an integral part of the overall Austrian welfare state structures, which is characterized by a high degree of public intervention and social protection mechanisms [55]. However, social services are closely aligned with achievements through gainful employment. In 2014, spendings on public social welfare benefit accounted for of 30% of annual economic value added. Healthcare comprised one quarter of total welfare spending, while another 9% (€ 9.2 bn in 2014) was on families. The proportion of spendings on social exclusion and housing was, however, only 2% [55]. The largest proportion of the family benefits was spent on cash benefits. Some identified cash benefits, such as family allowances, tax credits for childcare and childcare allowances, are universal transfer payments, meaning that they are independent of gainful activity and income. Other types are however dependent on employment and income (maternity allowances around childbirth), while a third category of cash benefits is means-tested, meaning that eligibility is linked to specific needs in the absence of own economic resources. Compared to international benchmarks, expenditure for in-kind benefits (e.g. subsidies for childcare facilities or family services) is low in Tyrol, in comparison to cash-benefits [56]. The regional and local governemts are the key providers of in-kind benefits. Their expenditure totalled € 2.5 bn in 2014 [55].

In Tyrol, no data are available on overall spending on mental health care. However, findings from previous research demonstrate that the capacity of publicly funded services is more likely to be restricted in the mental health area compared to physical health services. Consequently, patients with a mental illness will likely face private out-of-pocket costs. For example, in relation to need, there is a low number of child and adolescent psychiatrists who hold a contract with the health insurance in the outpatient sector. Likewise, access to publicly funded psychotherapy services is restricted, due to limited capacities and patients often need to consult a private therapist. Limited availability to services is particularly noted for child and adolescent mental healthcare [53–55].

This is supported by the observations of interviewees.

*“The parents have to pay themselves. And then you have to look constantly for donations or something, because a lot of people can’t afford it at all. They are often single parents (...), minimum pension, that’s our task, that we then look where we can get the money so that they [children] can visit this group.”* (interview 17).

Finally, regarding resources, the interviews also demonstrate that lack of (perceived) time resources among the adult mental health staff may contribute to the barriers of asking patients about parenting and thus for identifying affected children.

*“Doctors … are very often reluctant. They say they don’t have the time, they can’t speak with the families.”* (*interview 10*).

## Discussion

In this paper we aimed to develop an in-depth understanding of the situation facing children with a parent with a mental illness in Tyrol, Austria, in terms of identification and support. This knowledge was used to inform the next steps of the project, which were to design the co-development process for developing the practice approaches, together with local stakeholders, and to implement the practices in the local organisations. The conceptual matrix by Watson et al. [32] was used to systematically identify external context factors that may influence the co-development and implementation process.

While the precise prevalence of parental mental illness and their children in Tyrol remains unknown, administrative data on mental health benefit uptake demonstrated that a substantial number of parents may be reached via the primary health care system (e.g. the general practitioner), and only 5% via psychiatric secondary care (inpatient hospital setting). However, those in inpatient care could be considered the most severely ill parents, whose support needs for themselves and their children may consequently be higher. Furthermore, the results suggest that more mothers than fathers with a mental illness could be reached via adult mental health care overall, while the gender-difference is less pronounced in the hospital setting.

Although Austrian public expenditure for families and children could be considered as generally high compared to other countries [56], the data have shown that there are extremelly limited services available that target children who live with a parent with a mental disorder. One reason may be that the largest share of family expenditure are cash benefits rather than benefits in-kind. However, among the existing services, there is a potential of professional resources and an array of services available that may be accessed and coordinated for addressing different types of needs individual children may have. To some extent, specific cash benefits that are available in Tyrol may serve as an additional resource of support. Yet, while this variety offers flexibility for organising individualised and needs-based support, results on the relational climate and the funding and economic climate suggest that considerable challenges for coordination and organising individualised support could be faced, due to various funding and legal arrangements. Importantly, several services may have limited access for families due to referral pathways or geographical variations. As shown in the results on ‘target population’, many services for children are restricted to children having problems already, while our approach focuses on low-threshold early intervention, where existing services could be inappropriate or not accessible for our target group of healthy children. Targeting healthy children may, in the long run, reduce child and adolescent hospital resource use, or resources required for additional support in school, which has been shown to be higher for children who have a parent with a mental disorder than for children with parents who are mentally healthy [12]. As potentially relevant existing services or new services for supporting the children are likely part of the social sector (see results on ‘relational climate’), stable and sufficient funding seems to be difficult to achieve under the current legal and funding conditions. Moreover, the important role of the local and regional government in the current legal structure suggests that it will be important to address local politics in implementation and sustainability strategies. A facilitating factor for the successful and sustainable implementation of the planned practice approaches seems therefore to strive for integrating the practices into the existing routine processes (e.g. integrating identification of children into the routine assessment of patient admissions in adult mental health), rather than creating new identification and support services that may not have sustainable funding beyond the duration of the project.

Furthermore, at the level of professionals and the target population, expert observations have suggested fear and limited help seeking among affected parents and / or their children and feelings of shame will need to be addressed within the topic of parenting in both the parent with a mental illness, and the treating professionals. Alongside this, addressing structural and legal conditions that fuel those fears will be critical. Furthermore it has become clear from our results (e.g. see ‘professional influence’ or ‘relational climate’) that a family-focused care philosophy, and parenting in general, has so far not been part of the treatment concept in a standardized form in adult mental health and thus, professionals may need support to engage in these conversations with parents in a safe and non-judgemental way.

As has been outlined in the introduction, our support concept focuses on mobilising and utilising informal resources around the individual children / families before more formal and professional support is sought. Accordingly, our results indicate concerns among experts and people with lived experience on the potential medicalistion of children’s lives. Thus, while all children affected may need general support during episodes of their parent’s mental illness, a proportion of children are otherwise healthy and may not necessarily need professional care. Parameters for demography and socio-economics identified in our analysis indicate that Tyrol is a conservative region in terms of family composition and education / employment characteristics (e.g. fewer resources for childcare than in other parts of Austria [57], high rate of part-time working females). Furthermore, Catholic religion plays an important role and the strong link between Catholicism and charity and the traditional “christian-social” stance may have an influence on the population’s motivation for informal support and voluntary work. Therefore, informal supports and a community system could still play a big role in everday life for families in this region. Not least, we identified a number of activities within the voluntary sector (e.g. self-help groups) which could be potential resources for families. This implies that it may be feasible in our pilot region for families and children to utilise informal resources for support alongside more formal support structures. However, stigma and secrecy could be more challenging than in regions with more progressive character. The Austrian health targets, as well as the child and adolescent health strategy, may be utilized at the national policy level to foster implementation of the practice approaches, however, at the regional level, political backing is less pronounced and the topic has not been defined as an explicit strategic goal at the administrative planning level so far.

A limitation of this work is that contextual information collected and analysed is likely to be incomplete: The published data used for mapping the services may not represent changes in the service infrastructure that may have happened after the publication dates. Additionally, there are some limitations related to administrative data used regarding validity of the information on diagnoses of mental disorders. Furthermore, they do not cover the full range of mental health services available. However, our results and data collected were reviewed by regional experts at the service planning and funding level, who have detailed knowledge on available services within the region, and findings were presented to an open forum with representatives from service providers in the health, social and educational sector as well as people with lived experience including the project’s competence group (see introduction) for comments.

Recruitment of participants to be interviewed was based on the mapping of services, in which we attempted to represent all the service settings, as well as people with lived experience. However, although we conducted seventeen interviews and reached data saturation, we had limited number of participants per sector, which may have limited the diversity of views within these sectors. Additionally, qualitative methods do not aim to generalise and interview findings have provided rich and detailed perspectives from a range of sectors which helped in explaining other data sources, and also informing next project steps. That said, we found common themes across interviews and sectors. According to accepted practice in qualitative research, authors’ previous experience and background should also be considered when interpreting results. JP is an Australian native qualitative health researcher with expertise in healthcare interaction research. She relocated to Tyrol to conduct this research and currently has limited German skills. IZ is an Austrian health economist and health services researcher with advanced knowledge on the service landscape. MG is an implementation scientist with advanced knowledge in the area of supporting COPMIs in Australia and other countries and very limited German skills. This process of reflexivity allowed potential preconceptions to be challenged within this international, interdisciplinary group.

Authors of the applied conceptual framework on the external context note, the conceptual matrix may not cover all aspects of external context (e.g. physical environment such as topography) and the categories are not entirely mutually exclusive [32]. While we gained valuable insights into the current situation by this systematic approach, the context information may therefore to some extent still be incomplete.

## Conclusion

Identifying and supporting children who have a parent with a mental disorder in a standardised way can be defined as complex intervention. For developing specific practice approaches and successful implementation in our pilot region, extensive context knowledge is required. The systematic analysis of the external implementation context demonstrated that key stakeholders are aware of unmet needs, and that the societal structures and the current services provide a rich resource for improving identification and supporting of the children. However, considerable coordination and behaviour change efforts will be required, due to the fragmentation of the system and professional cultures. The insights into the context of supporting children have been of high value for designing the co-developing process during which researchers, practitioners and people with lived-experience were jointly developing practice approaches for their specific organisations in order to improve the situation for affected children and their families.

## Supplementary information

**Additional file 1: Supplementary file 1:** Interview guide.

## Data Availability

The interview datasets and the administrative datasets generated and/or analysed during the current study are not publicly available due to privacy and data protection law reasons. The natational and regional statistics as well as published materials used for the paper are are available from the corresponding author on reasonable request.

## Abbreviations

COPMI: Children of parents with a mental illness
ICD: International classification of diseases
GDP: Gross domestic product
