# Supplementary material for: Supporting children who have a parent with a mental illness in Tyrol: a situational analysis for informing co-development and implementation of practice changes
Source: BMC Health Serv Res. 2020 Apr 19;20:326. doi: 10.1186/s12913-020-05184-8 (PMC7168853; doi:10.1186/s12913-020-05184-8)
Supplement: Supplementary file 1 — Additional file 1: Supplementary file 1: Interview guide. [file 12913_2020_5184_MOESM1_ESM.docx]

*The Village* - Discussion Schedule (topic guide) for interviews with expert practitioners in Tyrol

SECTION 1: INTRODUCTION

- **Thank the person for participating in the research**
- **Provide the person with brief outline of the research and the aims of the interview** (they will have already had information from our invite and some may know project from info event or kick-off; so this can be very brief depending on interviewee; aim of project: improve identification and support of COPMI; develop (with stakeholders), implement and evaluate practice approaches; aim of interviews: getting to know the situation for children in Tyrol who have a parent with mental disorder and their families; learning about the challenges in daily practice from those experts who are involved and getting a better understanding of how we might be able to support these challenges through our project) (we are looking at the impact of early identification of children of parents with a mental illness and how to strengthen their support system to improve outcomes for these children and their families)
- **Explain a bit about yourself** (for example take role of naïve interviewer or expert from another field)
- **Remind person of informed consent** (the person will have read and signed informed consent form – if not yet signed ensure that person signs before interview or gives verbal consent and emphasise that we will still need to get written consent afterwards; this includes information about data recording; their right to withdraw from the research at any time and whether they want to be named or remain anonymous in publications)
- **Explain the interview process to person** (i.e. the number of areas to discuss and overall duration 45 minutes; what will happen after the interview; when the recording starts and ends; interviewees have received questions in advance)
- **Provide participant with an opportunity to ask questions about the research and interview process** (if they ask about workshops –we will be asking interested stakeholders to work with us in these workshops (6 in total from Oct-March) to develop the identification and collaborative care approaches that stakeholders believe will work in their context).
- **Inform them you will start recording now**

[Note: exact order will be up to researcher]

**TURN ON RECORDER**

SECTION 2: QUESTIONS

**Topic 1: role and current practice**

**Explain to participant that you would like to find out more about their role in the organisation as well as how they think COPMI are generally supported in Tyrol/Austria** **and about the role the organisation has in relation to the topic**

1. Could we begin with you explaining a little about your organization and your role within that organization?
2. What do you think the situation might be for children who have a parent with mental illness? What do you know of the situation for these children in Tyrol specifically?

*Prompt*

- 1. Do you think there are services to support these children in Tyrol? What services may be available for their parents?
  2. What do you know, from your experience, of the barriers to identification/access to these children?

1. What current involvement, if any, do you or your service have in supporting families /children where a parent has a mental illness? [*depends on interview partner; some are actively involved others only marginally or do not see themselves as relevant*]

*Prompts*

- 1. How does the service look like? [*ask for target group, financing, payers, sustainability of service etc.]*
  2. (How) does your organization currently identify parenting status, mental health status and child well-being information? [*depends on interviewee – only relevant for those who directly provide services, some interviewees are representatives of payers or coordinators]*
  3. How does your current organisation work with other services to support children of parents with a mental illness? What gets in the way of you working with other services to support these children and their families? *{want to know about collaborative care}*
  4. How does your organization typically document your processes and practices with clients? *[this is to find out about potential data that we may need for evaluation]*

1. What experience has your organization had working with other community and informal supports for children from vulnerable families?
2. What do you know of any other support that is provided to children of parents with a mental illness or their families in Tyrol or Austria?

**Topic 2: (workforce) changes and barriers to overcome**

**Explain to participant that you would like to get to know the barriers that occur for supporting COPMI and their families and that you would also like to learn what may support organisations in engaging in the topic**

1. What do you think might make it difficult for professionals to identify and support the needs of children of parents with mental illness in your organisation/in Tyrol? *[ask for financial, organisation, political, personnel, etc. conditions]*
   1. How do you think we could work to address these barriers?
2. What do you believe might make it difficult for professionals to work with other professionals and the family to support the needs of children of parents with a mental illness?
   1. What might help address these barriers?
3. What would you need as an organisation so that your staff could engage more in identifying and supporting COPMI? (Alternative: /what would organisations need so that their staff could engage more in identifying and supporting COPMI?)

Prompts:

- 1. How do you think we could enhance the practice of professionals to identify and support children of parents with mental illness in Austria? What might be the ways we could engage workers to change their practice to support COPMI?
  2. What has worked before, from your experience, to support professionals to undertake a new practice?

1. Where do you see opportunities for changing practice towards identifying and supporting COPMI?

**Topic 3**: general feedback

**Explain that you would like to complete the interview with some general feedback**

1. Overall, what key messages do you have for the village project attempting to improve practice of identifying and providing support for COPMI in Tyrol based services?
2. Is there anything else you would like to raise?

Thank you for participating in the interview!

END RECORDING

Remind them about interview process (i.e. that we will get in contact with them with a transcript of the interviews that they can comment on; they can withdraw information up until data analysis; also plans for publication)
